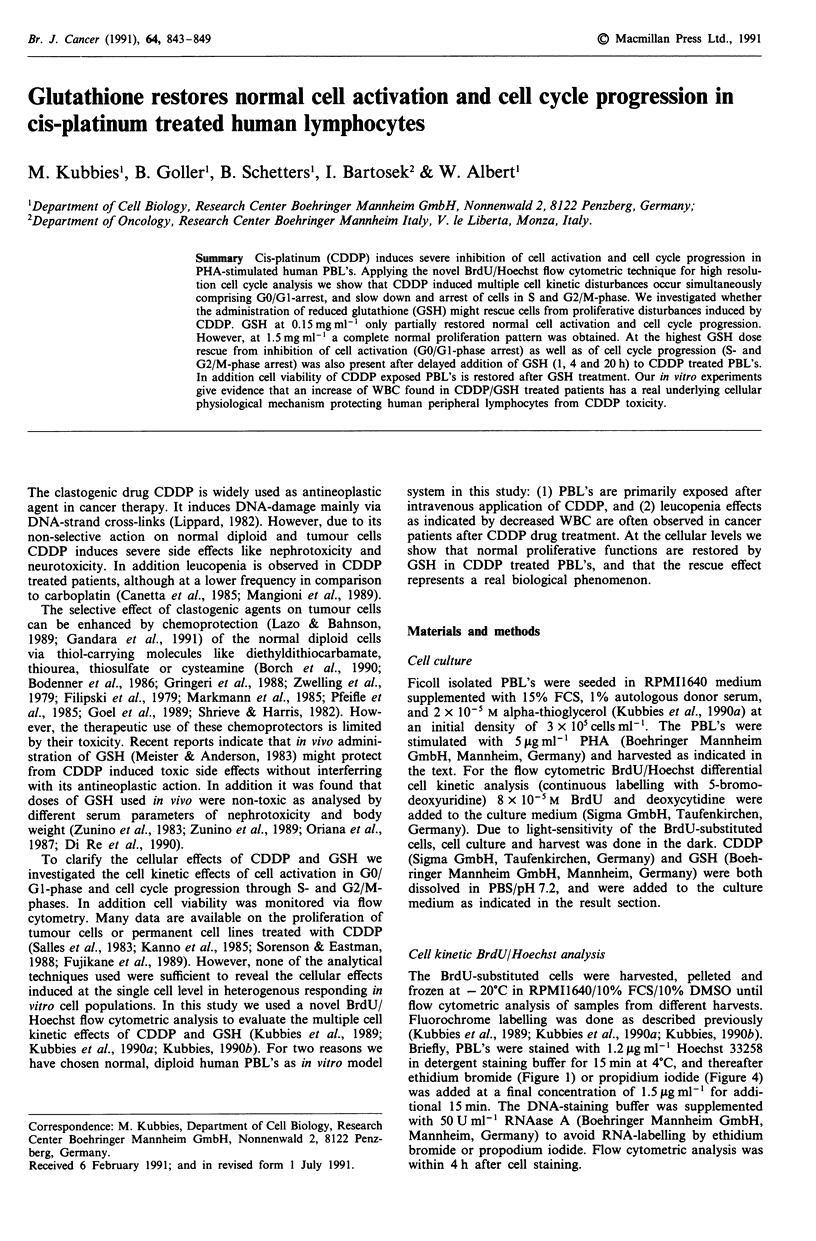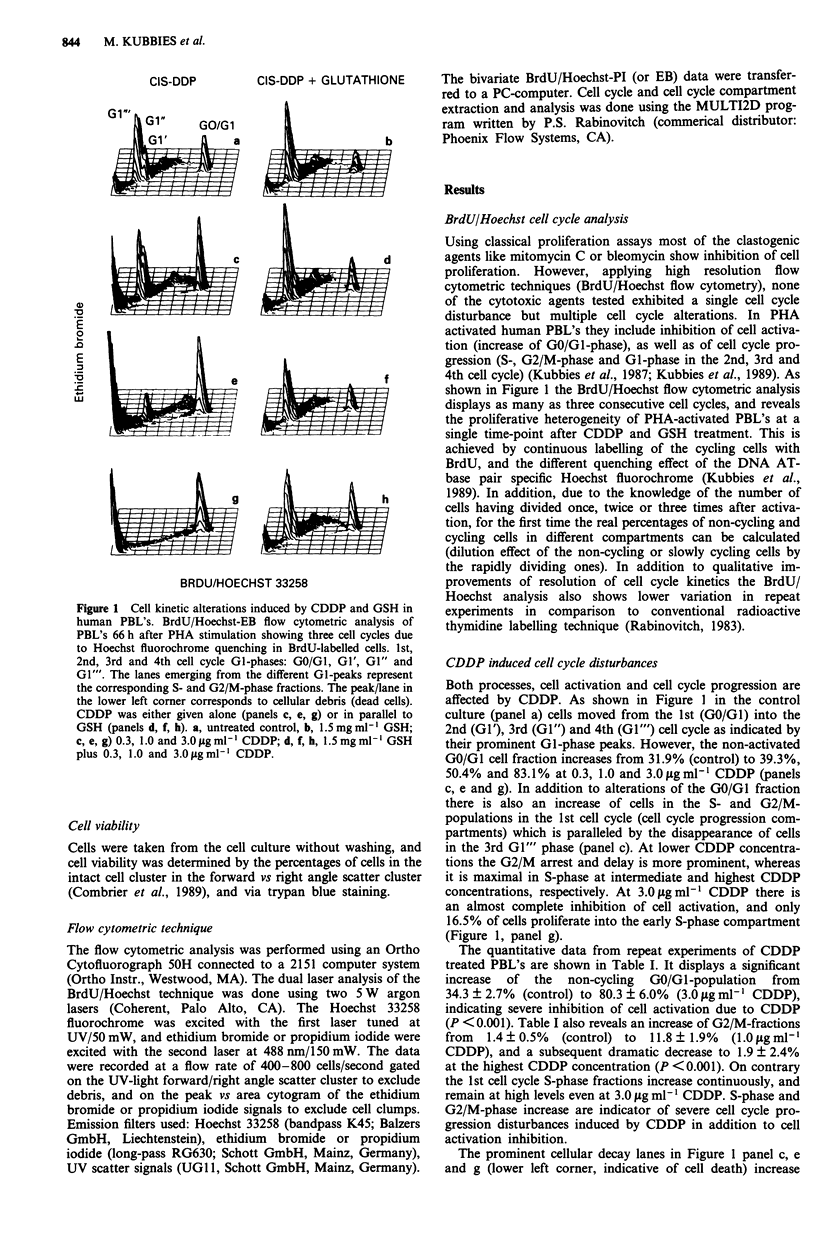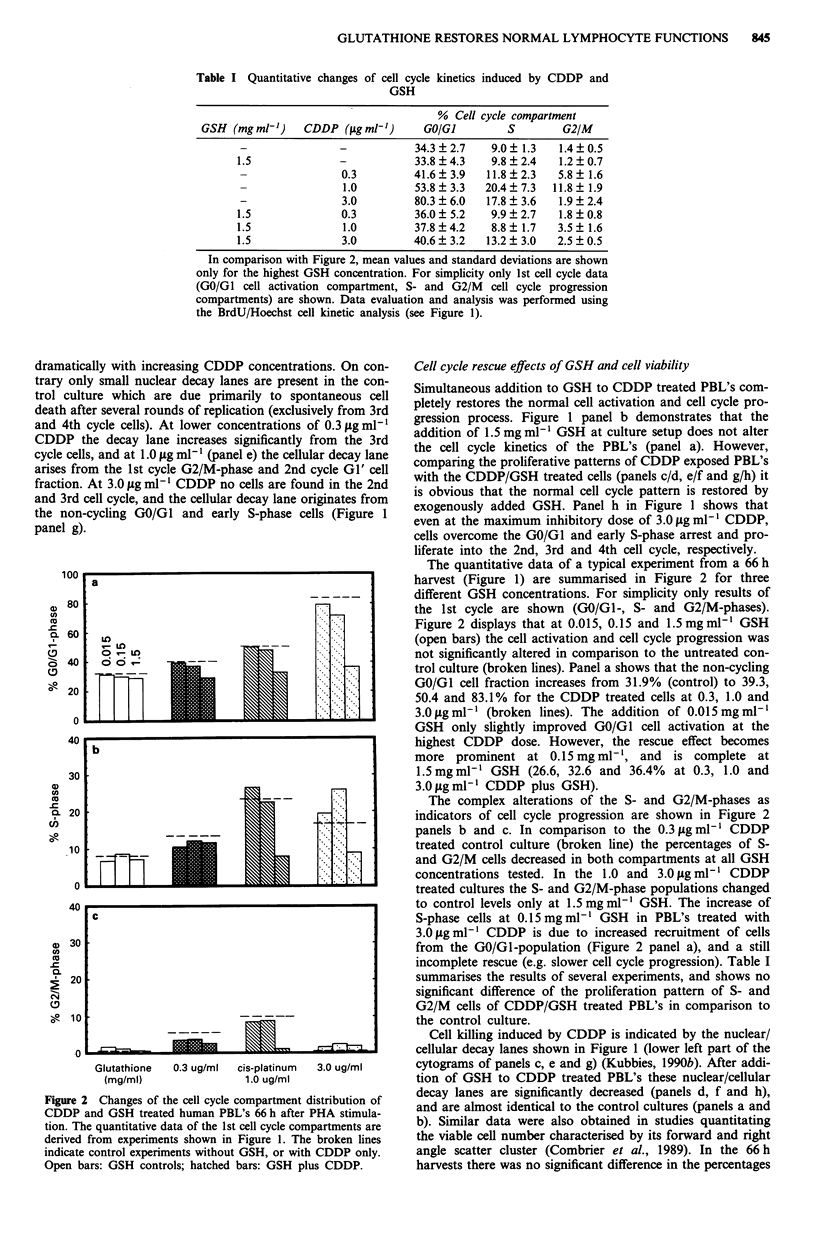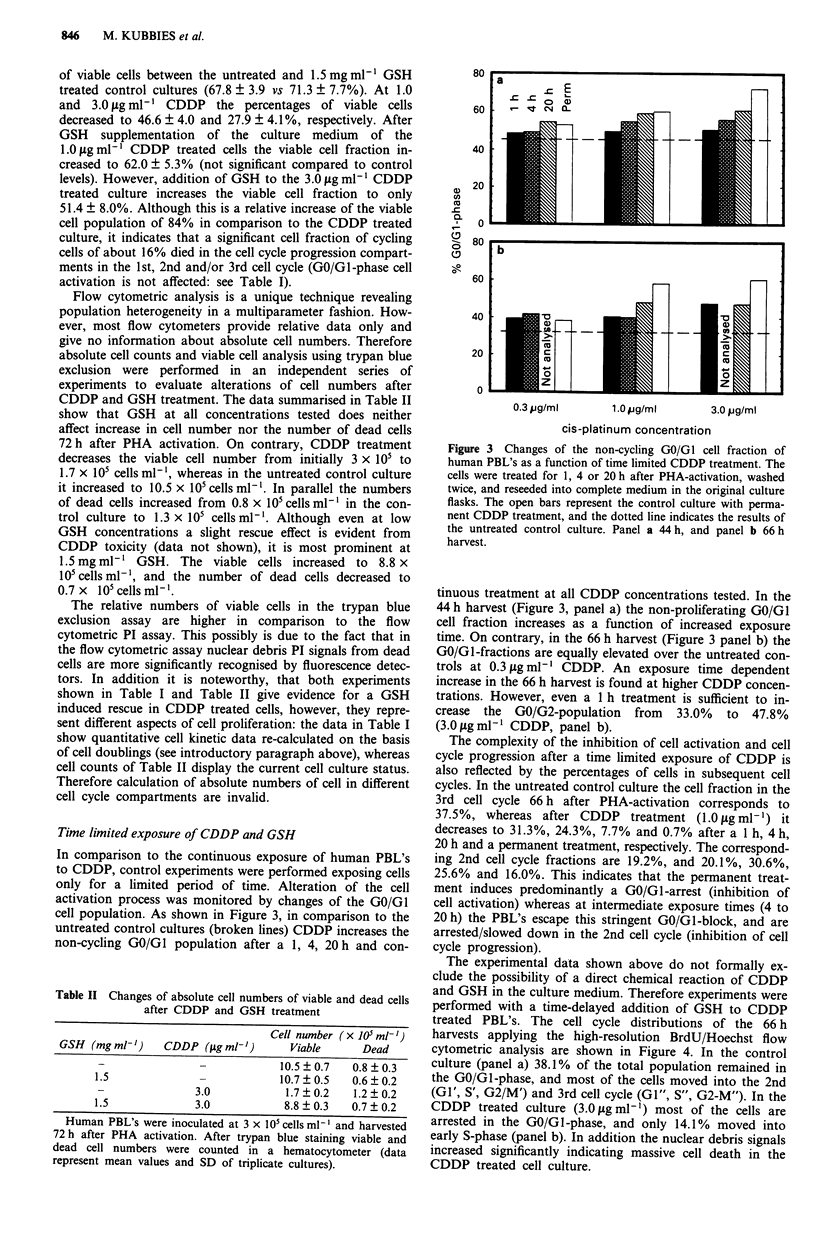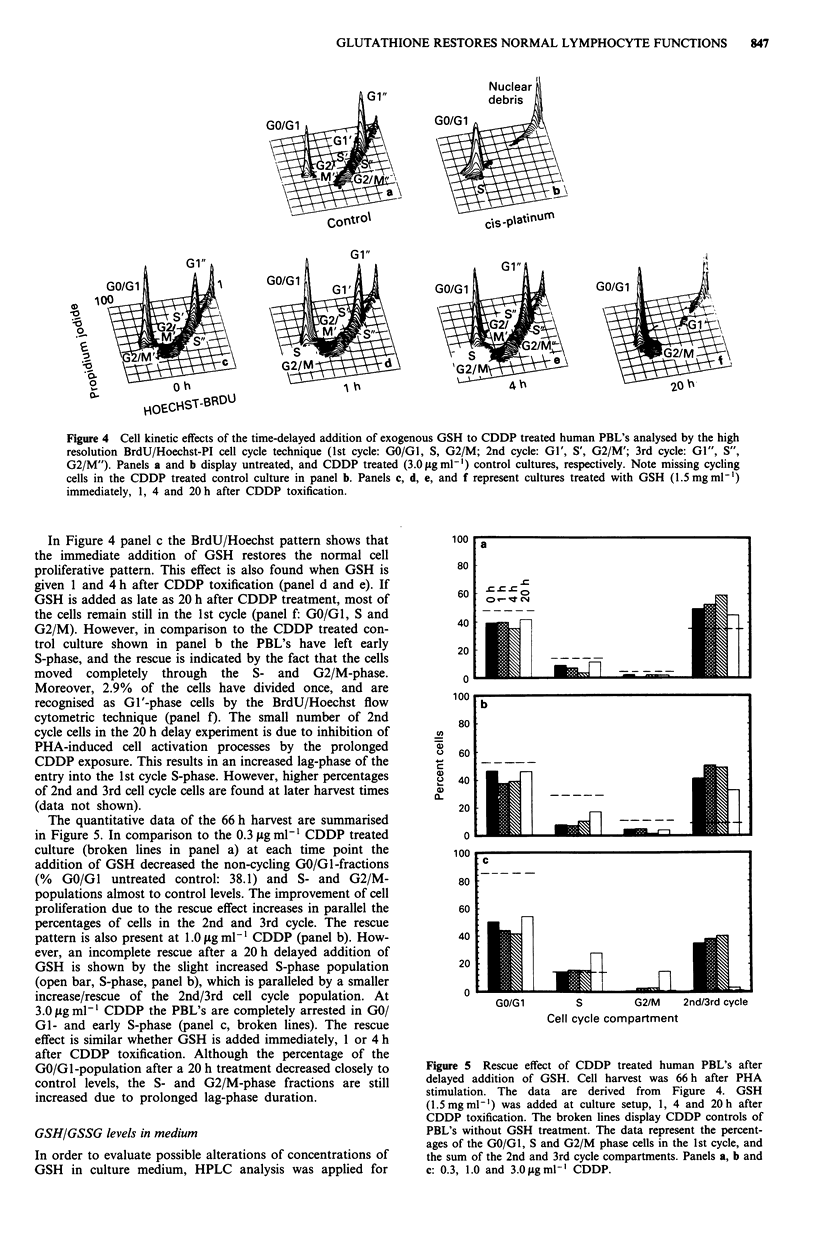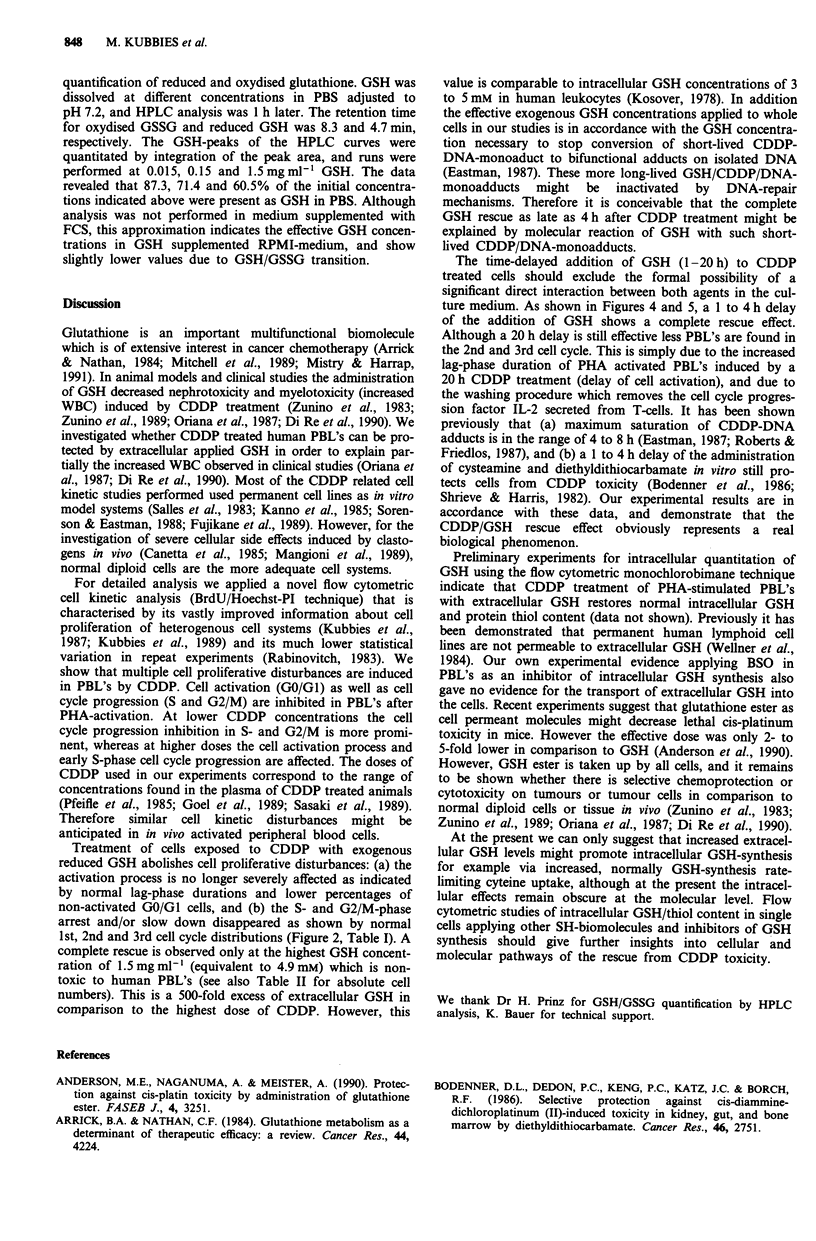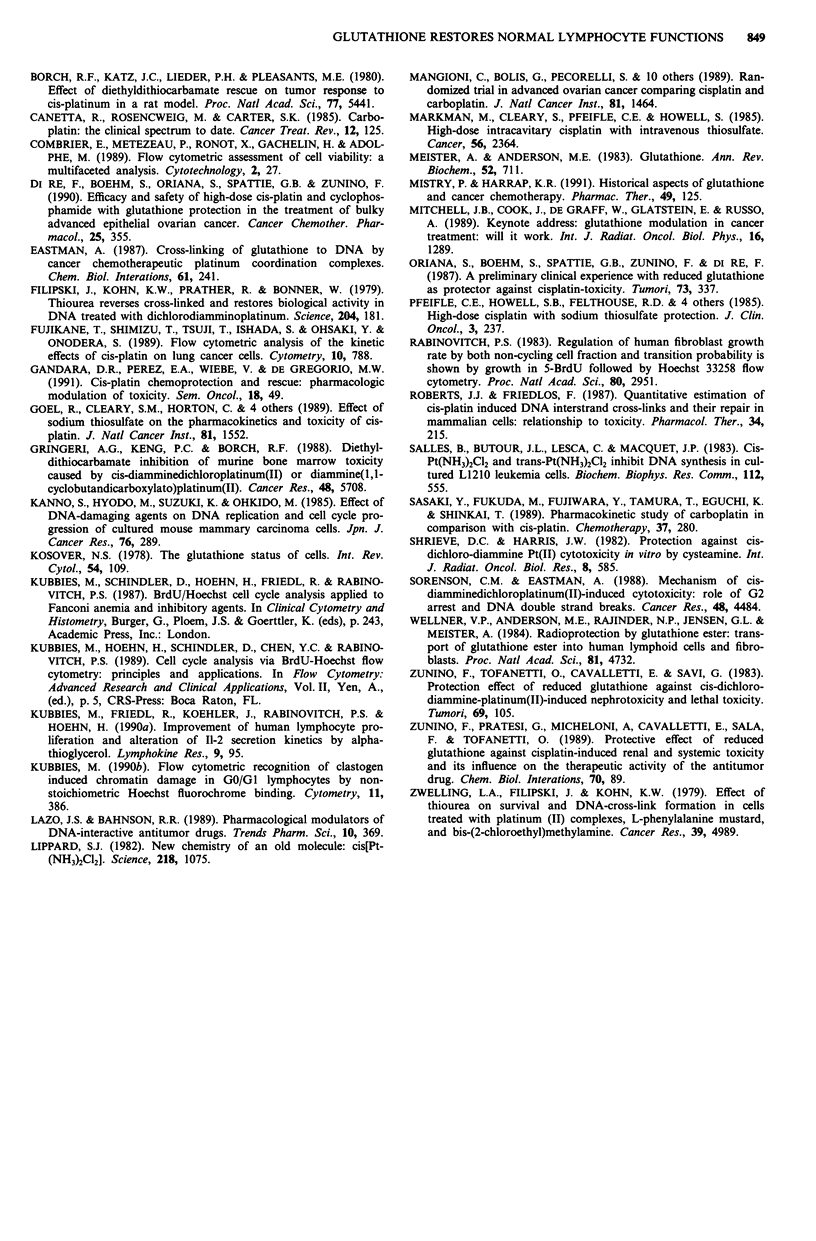# Glutathione restores normal cell activation and cell cycle progression in cis-platinum treated human lymphocytes.

**DOI:** 10.1038/bjc.1991.411

**Published:** 1991-11

**Authors:** M. Kubbies, B. Goller, B. Schetters, I. Bartosek, W. Albert

**Affiliations:** Department of Cell Biology, Research Center Boehringer Mannheim GmbH, Penzberg, Germany.

## Abstract

Cis-platinum (CDDP) induces severe inhibition of cell activation and cell cycle progression in PHA-stimulated human PBL's. Applying the novel BrdU/Hoechst flow cytometric technique for high resolution cell cycle analysis we show that CDDP induced multiple cell kinetic disturbances occur simultaneously comprising G0/G1-arrest, and slow down and arrest of cells in S and G2/M-phase. We investigated whether the administration of reduced glutathione (GSH) might rescue cells from proliferative disturbances induced by CDDP. GSH at 0.15 mg ml-1 only partially restored normal cell activation and cell cycle progression. However, at 1.5 mg ml-1 a complete normal proliferation pattern was obtained. At the highest GSH dose rescue from inhibition of cell activation (G0/G1-phase arrest) as well as of cell cycle progression (S- and G2/M-phase arrest) was also present after delayed addition of GSH (1, 4 and 20 h) to CDDP treated PBL's. In addition cell viability of CDDP exposed PBL's is restored after GSH treatment. Our in vitro experiments give evidence that an increase of WBC found in CDDP/GSH treated patients has a real underlying cellular physiological mechanism protecting human peripheral lymphocytes from CDDP toxicity.


Br. J. Cancer (1991), 64, 843-849 © Macmillan Press Ltd., 1991

### Glutathione restores normal cell activation and cell cycle progression in cis-platinum treated human lymphocytes

M. Kubbies[1], B. Goller[1], B. Schetters[1], I. Bartosek[2] & W. Albert[1]

[1]*Department of Cell Biology, Research Center Boehringer Mannheim GmbH, Nonnenwald 2, 8122 Penzberg, Germany;*
[2]*Department of Oncology, Research Center Boehringer Mannheim Italy, V. le Liberta, Monza, Italy.*

**Summary** Cis-platinum (CDDP) induces severe inhibition of cell activation and cell cycle progression in PHA-stimulated human PBL's. Applying the novel BrdU/Hoechst flow cytometric technique for high resolution cell cycle analysis we show that CDDP induced multiple cell kinetic disturbances occur simultaneously comprising G0/G1-arrest, and slow down and arrest of cells in S and G2/M-phase. We investigated whether the administration of reduced glutathione (GSH) might rescue cells from proliferative disturbances induced by CDDP. GSH at 0.15 mg ml$^{-1}$ only partially restored normal cell activation and cell cycle progression. However, at 1.5 mg ml$^{-1}$ a complete normal proliferation pattern was obtained. At the highest GSH dose rescue from inhibition of cell activation (G0/G1-phase arrest) as well as of cell cycle progression (S- and G2/M-phase arrest) was also present after delayed addition of GSH (1, 4 and 20 h) to CDDP treated PBL's. In addition cell viability of CDDP exposed PBL's is restored after GSH treatment. Our *in vitro* experiments give evidence that an increase of WBC found in CDDP/GSH treated patients has a real underlying cellular physiological mechanism protecting human peripheral lymphocytes from CDDP toxicity.

The clastogenic drug CDDP is widely used as antineoplastic agent in cancer therapy. It induces DNA-damage mainly via DNA-strand cross-links (Lippard, 1982). However, due to its non-selective action on normal diploid and tumour cells CDDP induces severe side effects like nephrotoxicity and neurotoxicity. In addition leucopenia is observed in CDDP treated patients, although at a lower frequency in comparison to carboplatin (Canetta *et al.*, 1985; Mangioni *et al.*, 1989).

The selective effect of clastogenic agents on tumour cells can be enhanced by chemoprotection (Lazo & Bahnson, 1989; Gandara *et al.*, 1991) of the normal diploid cells via thiol-carrying molecules like diethyldithiocarbamate, thiourea, thiosulfate or cysteamine (Borch *et al.*, 1990; Bodenner *et al.*, 1986; Gringeri *et al.*, 1988; Zwelling *et al.*, 1979; Filipski *et al.*, 1979; Markmann *et al.*, 1985; Pfeifle *et al.*, 1985; Goel *et al.*, 1989; Shrieve & Harris, 1982). However, the therapeutic use of these chemoprotectors is limited by their toxicity. Recent reports indicate that *in vivo* administration of GSH (Meister & Anderson, 1983) might protect from CDDP induced toxic side effects without interferring with its antineoplastic action. In addition it was found that doses of GSH used *in vivo* were non-toxic as analysed by different serum parameters of nephrotoxicity and body weight (Zunino *et al.*, 1983; Zunino *et al.*, 1989; Oriana *et al.*, 1987; Di Re *et al.*, 1990).

To clarify the cellular effects of CDDP and GSH we investigated the cell kinetic effects of cell activation in G0/G1-phase and cell cycle progression through S- and G2/M-phases. In addition cell viability was monitored via flow cytometry. Many data are available on the proliferation of tumour cells or permanent cell lines treated with CDDP (Salles *et al.*, 1983; Kanno *et al.*, 1985; Sorenson & Eastman, 1988; Fujikane *et al.*, 1989). However, none of the analytical techniques used were sufficient to reveal the cellular effects induced at the single cell level in heterogenous responding *in vitro* cell populations. In this study we used a novel BrdU/Hoechst flow cytometric analysis to evaluate the multiple cell kinetic effects of CDDP and GSH (Kubbies *et al.*, 1989; Kubbies *et al.*, 1990*a*; Kubbies, 1990*b*). For two reasons we have chosen normal, diploid human PBL's as *in vitro* model system in this study: (1) PBL's are primarily exposed after intravenous application of CDDP, and (2) leucopenia effects as indicated by decreased WBC are often observed in cancer patients after CDDP drug treatment. At the cellular levels we show that normal proliferative functions are restored by GSH in CDDP treated PBL's, and that the rescue effect represents a real biological phenomenon.

Correspondence: M. Kubbies, Department of Cell Biology, Research Center Boehringer Mannheim GmbH, Nonnenwald 2, 8122 Penzberg, Germany.
Received 6 February 1991; and in revised form 1 July 1991.

### Materials and methods

### *Cell culture*

Ficoll isolated PBL's were seeded in RPMI1640 medium supplemented with 15% FCS, 1% autologous donor serum, and $2 \times 10^{-5}$ M alpha-thioglycerol (Kubbies *et al.*, 1990*a*) at an initial density of $3 \times 10^5$ cells ml$^{-1}$. The PBL's were stimulated with 5 μg ml$^{-1}$ PHA (Boehringer Mannheim GmbH, Mannheim, Germany) and harvested as indicated in the text. For the flow cytometric BrdU/Hoechst differential cell kinetic analysis (continuous labelling with 5-bromodeoxyuridine) $8 \times 10^{-5}$ M BrdU and deoxycytidine were added to the culture medium (Sigma GmbH, Taufenkirchen, Germany). Due to light-sensitivity of the BrdU-substituted cells, cell culture and harvest was done in the dark. CDDP (Sigma GmbH, Taufenkirchen, Germany) and GSH (Boehringer Mannheim GmbH, Mannheim, Germany) were both dissolved in PBS/pH 7.2, and were added to the culture medium as indicated in the result section.

### *Cell kinetic BrdU/Hoechst analysis*

The BrdU-substituted cells were harvested, pelleted and frozen at − 20°C in RPMI1640/10% FCS/10% DMSO until flow cytometric analysis of samples from different harvests. Fluorochrome labelling was done as described previously (Kubbies *et al.*, 1989; Kubbies *et al.*, 1990*a*; Kubbies, 1990*b*). Briefly, PBL's were stained with 1.2 μg ml$^{-1}$ Hoechst 33258 in detergent staining buffer for 15 min at 4°C, and thereafter ethidium bromide (Figure 1) or propidium iodide (Figure 4) was added at a final concentration of 1.5 μg ml$^{-1}$ for additional 15 min. The DNA-staining buffer was supplemented with 50 U ml$^{-1}$ RNAase A (Boehringer Mannheim GmbH, Mannheim, Germany) to avoid RNA-labelling by ethidium bromide or propodium iodide. Flow cytometric analysis was within 4 h after cell staining.

**Figure 1** Cell kinetic alterations induced by CDDP and GSH in human PBL's. BrdU/Hoechst-EB flow cytometric analysis of PBL's 66 h after PHA stimulation showing three cell cycles due to Hoechst fluorochrome quenching in BrdU-labelled cells. 1st, 2nd, 3rd and 4th cell cycle G1-phases: G0/G1, G1', G1'' and G1'''. The lanes emerging from the different G1-peaks represent the corresponding S- and G2/M-phase fractions. The peak/lane in the lower left corner corresponds to cellular debris (dead cells). CDDP was either given alone (panels c, e, g) or in parallel to GSH (panels **d**, **f**, **h**). **a**, untreated control, **b**, 1.5 mg ml$^{-1}$ GSH; **c**, **e**, **g**) 0.3, 1.0 and 3.0 μg ml$^{-1}$ CDDP; **d**, **f**, **h**, 1.5 mg ml$^{-1}$ GSH plus 0.3, 1.0 and 3.0 μg ml$^{-1}$ CDDP.

*Cell viability*

Cells were taken from the cell culture without washing, and cell viability was determined by the percentages of cells in the intact cell cluster in the forward *vs* right angle scatter cluster (Combrier *et al.*, 1989), and via trypan blue staining.

*Flow cytometric technique*

The flow cytometric analysis was performed using an Ortho Cytofluorograph 50H connected to a 2151 computer system (Ortho Instr., Westwood, MA). The dual laser analysis of the BrdU/Hoechst technique was done using two 5 W argon lasers (Coherent, Palo Alto, CA). The Hoechst 33258 fluorochrome was excited with the first laser tuned at UV/50 mW, and ethidium bromide or propidium iodide were excited with the second laser at 488 nm/150 mW. The data were recorded at a flow rate of 400–800 cells/second gated on the UV-light forward/right angle scatter cluster to exclude debris, and on the peak *vs* area cytogram of the ethidium bromide or propidium iodide signals to exclude cell clumps. Emission filters used: Hoechst 33258 (bandpass K45; Balzers GmbH, Liechtenstein), ethidium bromide or propidium iodide (long-pass RG630; Schott GmbH, Mainz, Germany), UV scatter signals (UG11, Schott GmbH, Mainz, Germany).

The bivariate BrdU/Hoechst-PI (or EB) data were transferred to a PC-computer. Cell cycle and cell cycle compartment extraction and analysis was done using the MULTI2D program written by P.S. Rabinovitch (commerical distributor: Phoenix Flow Systems, CA).

### Results

*BrdU/Hoechst cell cycle analysis*

Using classical proliferation assays most of the clastogenic agents like mitomycin C or bleomycin show inhibition of cell proliferation. However, applying high resolution flow cytometric techniques (BrdU/Hoechst flow cytometry), none of the cytotoxic agents tested exhibited a single cell cycle disturbance but multiple cell cycle alterations. In PHA activated human PBL's they include inhibition of cell activation (increase of G0/G1-phase), as well as of cell cycle progression (S-, G2/M-phase and G1-phase in the 2nd, 3rd and 4th cell cycle) (Kubbies *et al.*, 1987; Kubbies *et al.*, 1989). As shown in Figure 1 the BrdU/Hoechst flow cytometric analysis displays as many as three consecutive cell cycles, and reveals the proliferative heterogeneity of PHA-activated PBL's at a single time-point after CDDP and GSH treatment. This is achieved by continuous labelling of the cycling cells with BrdU, and the different quenching effect of the DNA AT-base pair specific Hoechst fluorochrome (Kubbies *et al.*, 1989). In addition, due to the knowledge of the number of cells having divided once, twice or three times after activation, for the first time the real percentages of non-cycling and cycling cells in different compartments can be calculated (dilution effect of the non-cycling or slowly cycling cells by the rapidly dividing ones). In addition to qualitative improvements of resolution of cell cycle kinetics the BrdU/Hoechst analysis also shows lower variation in repeat experiments in comparison to conventional radioactive thymidine labelling technique (Rabinovitch, 1983).

*CDDP induced cell cycle disturbances*

Both processes, cell activation and cell cycle progression are affected by CDDP. As shown in Figure 1 in the control culture (panel a) cells moved from the 1st (G0/G1) into the 2nd (G1'), 3rd (G1'') and 4th (G1''') cell cycle as indicated by their prominent G1-phase peaks. However, the non-activated G0/G1 cell fraction increases from 31.9% (control) to 39.3%, 50.4% and 83.1% at 0.3, 1.0 and 3.0 μg ml$^{-1}$ CDDP (panels c, e and g). In addition to alterations of the G0/G1 fraction there is also an increase of cells in the S- and G2/M-populations in the 1st cell cycle (cell cycle progression compartments) which is paralleled by the disappearance of cells in the 3rd G1''' phase (panel c). At lower CDDP concentrations the G2/M arrest and delay is more prominent, whereas it is maximal in S-phase at intermediate and highest CDDP concentrations, respectively. At 3.0 μg ml$^{-1}$ CDDP there is an almost complete inhibition of cell activation, and only 16.5% of cells proliferate into the early S-phase compartment (Figure 1, panel g).

The quantitative data from repeat experiments of CDDP treated PBL's are shown in Table I. It displays a significant increase of the non-cycling G0/G1-population from 34.3 ± 2.7% (control) to 80.3 ± 6.0% (3.0 μg ml$^{-1}$ CDDP), indicating severe inhibition of cell activation due to CDDP ($P < 0.001$). Table I also reveals an increase of G2/M-fractions from 1.4 ± 0.5% (control) to 11.8 ± 1.9% (1.0 μg ml$^{-1}$ CDDP), and a subsequent dramatic decrease to 1.9 ± 2.4% at the highest CDDP concentration ($P < 0.001$). On contrary the 1st cell cycle S-phase fractions increase continuously, and remain at high levels even at 3.0 μg ml$^{-1}$ CDDP. S-phase and G2/M-phase increase are indicator of severe cell cycle progression disturbances induced by CDDP in addition to cell activation inhibition.

The prominent cellular decay lanes in Figure 1 panel c, e and g (lower left corner, indicative of cell death) increase

**Table I** Quantitative changes of cell cycle kinetics induced by CDDP and GSH

| | | *% Cell cycle compartment* | | |
|---|---|---|---|---|
| *GSH* ($mg\,ml^{-1}$) | *CDDP* ($\mu g\,ml^{-1}$) | *G0/G1* | *S* | *G2/M* |
| – | – | 34.3 ± 2.7 | 9.0 ± 1.3 | 1.4 ± 0.5 |
| 1.5 | – | 33.8 ± 4.3 | 9.8 ± 2.4 | 1.2 ± 0.7 |
| – | 0.3 | 41.6 ± 3.9 | 11.8 ± 2.3 | 5.8 ± 1.6 |
| – | 1.0 | 53.8 ± 3.3 | 20.4 ± 7.3 | 11.8 ± 1.9 |
| – | 3.0 | 80.3 ± 6.0 | 17.8 ± 3.6 | 1.9 ± 2.4 |
| 1.5 | 0.3 | 36.0 ± 5.2 | 9.9 ± 2.7 | 1.8 ± 0.8 |
| 1.5 | 1.0 | 37.8 ± 4.2 | 8.8 ± 1.7 | 3.5 ± 1.6 |
| 1.5 | 3.0 | 40.6 ± 3.2 | 13.2 ± 3.0 | 2.5 ± 0.5 |

In comparison with Figure 2, mean values and standard deviations are shown only for the highest GSH concentration. For simplicity only 1st cell cycle data (G0/G1 cell activation compartment, S- and G2/M cell cycle progression compartments) are shown. Data evaluation and analysis was performed using the BrdU/Hoechst cell kinetic analysis (see Figure 1).

dramatically with increasing CDDP concentrations. On contrary only small nuclear decay lanes are present in the control culture which are due primarily to spontaneous cell death after several rounds of replication (exclusively from 3rd and 4th cycle cells). At lower concentrations of 0.3 $\mu g\,ml^{-1}$ CDDP the decay lane increases significantly from the 3rd cycle cells, and at 1.0 $\mu g\,ml^{-1}$ (panel e) the cellular decay lane arises from the 1st cycle G2/M-phase and 2nd cycle G1′ cell fraction. At 3.0 $\mu g\,ml^{-1}$ CDDP no cells are found in the 2nd and 3rd cell cycle, and the cellular decay lane originates from the non-cycling G0/G1 and early S-phase cells (Figure 1 panel g).

**Figure 2** Changes of the cell cycle compartment distribution of CDDP and GSH treated human PBL's 66 h after PHA stimulation. The quantitative data of the 1st cell cycle compartments are derived from experiments shown in Figure 1. The broken lines indicate control experiments without GSH, or with CDDP only. Open bars: GSH controls; hatched bars: GSH plus CDDP.

*Cell cycle rescue effects of GSH and cell viability*

Simultaneous addition to GSH to CDDP treated PBL's completely restores the normal cell activation and cell cycle progression process. Figure 1 panel b demonstrates that the addition of 1.5 $mg\,ml^{-1}$ GSH at culture setup does not alter the cell cycle kinetics of the PBL's (panel a). However, comparing the proliferative patterns of CDDP exposed PBL's with the CDDP/GSH treated cells (panels c/d, e/f and g/h) it is obvious that the normal cell cycle pattern is restored by exogenously added GSH. Panel h in Figure 1 shows that even at the maximum inhibitory dose of 3.0 $\mu g\,ml^{-1}$ CDDP, cells overcome the G0/G1 and early S-phase arrest and proliferate into the 2nd, 3rd and 4th cell cycle, respectively.

The quantitative data of a typical experiment from a 66 h harvest (Figure 1) are summarised in Figure 2 for three different GSH concentrations. For simplicity only results of the 1st cycle are shown (G0/G1-, S- and G2/M-phases). Figure 2 displays that at 0.015, 0.15 and 1.5 $mg\,ml^{-1}$ GSH (open bars) the cell activation and cell cycle progression was not significantly altered in comparison to the untreated control culture (broken lines). Panel a shows that the non-cycling G0/G1 cell fraction increases from 31.9% (control) to 39.3, 50.4 and 83.1% for the CDDP treated cells at 0.3, 1.0 and 3.0 $\mu g\,ml^{-1}$ (broken lines). The addition of 0.015 $mg\,ml^{-1}$ GSH only slightly improved G0/G1 cell activation at the highest CDDP dose. However, the rescue effect becomes more prominent at 0.15 $mg\,ml^{-1}$, and is complete at 1.5 $mg\,ml^{-1}$ GSH (26.6, 32.6 and 36.4% at 0.3, 1.0 and 3.0 $\mu g\,ml^{-1}$ CDDP plus GSH).

The complex alterations of the S- and G2/M-phases as indicators of cell cycle progression are shown in Figure 2 panels b and c. In comparison to the 0.3 $\mu g\,ml^{-1}$ CDDP treated control culture (broken line) the percentages of S- and G2/M cells decreased in both compartments at all GSH concentrations tested. In the 1.0 and 3.0 $\mu g\,ml^{-1}$ CDDP treated cultures the S- and G2/M-phase populations changed to control levels only at 1.5 $mg\,ml^{-1}$ GSH. The increase of S-phase cells at 0.15 $mg\,ml^{-1}$ GSH in PBL's treated with 3.0 $\mu g\,ml^{-1}$ CDDP is due to increased recruitment of cells from the G0/G1-population (Figure 2 panel a), and a still incomplete rescue (e.g. slower cell cycle progression). Table I summarises the results of several experiments, and shows no significant difference of the proliferation pattern of S- and G2/M cells of CDDP/GSH treated PBL's in comparison to the control culture.

Cell killing induced by CDDP is indicated by the nuclear/cellular decay lanes shown in Figure 1 (lower left part of the cytograms of panels c, e and g) (Kubbies, 1990*b*). After addition of GSH to CDDP treated PBL's these nuclear/cellular decay lanes are significantly decreased (panels d, f and h), and are almost identical to the control cultures (panels a and b). Similar data were also obtained in studies quantitating the viable cell number characterised by its forward and right angle scatter cluster (Combrier *et al.*, 1989). In the 66 h harvests there was no significant difference in the percentages of viable cells between the untreated and 1.5 mg ml$^{-1}$ GSH treated control cultures (67.8 ± 3.9 *vs* 71.3 ± 7.7%). At 1.0 and 3.0 μg ml$^{-1}$ CDDP the percentages of viable cells decreased to 46.6 ± 4.0 and 27.9 ± 4.1%, respectively. After GSH supplementation of the culture medium of the 1.0 μg ml$^{-1}$ CDDP treated cells the viable cell fraction increased to 62.0 ± 5.3% (not significant compared to control levels). However, addition of GSH to the 3.0 μg ml$^{-1}$ CDDP treated culture increases the viable cell fraction to only 51.4 ± 8.0%. Although this is a relative increase of the viable cell population of 84% in comparison to the CDDP treated culture, it indicates that a significant cell fraction of cycling cells of about 16% died in the cell cycle progression compartments in the 1st, 2nd and/or 3rd cell cycle (G0/G1-phase cell activation is not affected: see Table I).

Flow cytometric analysis is a unique technique revealing population heterogeneity in a multiparameter fashion. However, most flow cytometers provide relative data only and give no information about absolute cell numbers. Therefore absolute cell counts and viable cell analysis using trypan blue exclusion were performed in an independent series of experiments to evaluate alterations of cell numbers after CDDP and GSH treatment. The data summarised in Table II show that GSH at all concentrations tested does neither affect increase in cell number nor the number of dead cells 72 h after PHA activation. On contrary, CDDP treatment decreases the viable cell number from initially $3 \times 10^5$ to $1.7 \times 10^5$ cells ml$^{-1}$, whereas in the untreated control culture it increased to $10.5 \times 10^5$ cells ml$^{-1}$. In parallel the numbers of dead cells increased from $0.8 \times 10^5$ cells ml$^{-1}$ in the control culture to $1.3 \times 10^5$ cells ml$^{-1}$. Although even at low GSH concentrations a slight rescue effect is evident from CDDP toxicity (data not shown), it is most prominent at 1.5 mg ml$^{-1}$ GSH. The viable cells increased to $8.8 \times 10^5$ cells ml$^{-1}$, and the number of dead cells decreased to $0.7 \times 10^5$ cells ml$^{-1}$.

The relative numbers of viable cells in the trypan blue exclusion assay are higher in comparison to the flow cytometric PI assay. This possibly is due to the fact that in the flow cytometric assay nuclear debris PI signals from dead cells are more significantly recognised by fluorescence detectors. In addition it is noteworthy, that both experiments shown in Table I and Table II give evidence for a GSH induced rescue in CDDP treated cells, however, they represent different aspects of cell proliferation: the data in Table I show quantitative cell kinetic data re-calculated on the basis of cell doublings (see introductory paragraph above), whereas cell counts of Table II display the current cell culture status. Therefore calculation of absolute numbers of cell in different cell cycle compartments are invalid.

### *Time limited exposure of CDDP and GSH*

In comparison to the continuous exposure of human PBL's to CDDP, control experiments were performed exposing cells only for a limited period of time. Alteration of the cell activation process was monitored by changes of the G0/G1 cell population. As shown in Figure 3, in comparison to the untreated control cultures (broken lines) CDDP increases the non-cycling G0/G1 population after a 1, 4, 20 h and continuous treatment at all CDDP concentrations tested. In the 44 h harvest (Figure 3, panel a) the non-proliferating G0/G1 cell fraction increases as a function of increased exposure time. On contrary, in the 66 h harvest (Figure 3 panel b) the G0/G1-fractions are equally elevated over the untreated controls at 0.3 μg ml$^{-1}$ CDDP. An exposure time dependent increase in the 66 h harvest is found at higher CDDP concentrations. However, even a 1 h treatment is sufficient to increase the G0/G2-population from 33.0% to 47.8% (3.0 μg ml$^{-1}$ CDDP, panel b).

**Figure 3** Changes of the non-cycling G0/G1 cell fraction of human PBL's as a function of time limited CDDP treatment. The cells were treated for 1, 4 or 20 h after PHA-activation, washed twice, and reseeded into complete medium in the original culture flasks. The open bars represent the control culture with permanent CDDP treatment, and the dotted line indicates the results of the untreated control culture. Panel **a** 44 h, and panel **b** 66 h harvest.

The complexity of the inhibition of cell activation and cell cycle progression after a time limited exposure of CDDP is also reflected by the percentages of cells in subsequent cell cycles. In the untreated control culture the cell fraction in the 3rd cell cycle 66 h after PHA-activation corresponds to 37.5%, whereas after CDDP treatment (1.0 μg ml$^{-1}$) it decreases to 31.3%, 24.3%, 7.7% and 0.7% after a 1 h, 4 h, 20 h and a permanent treatment, respectively. The corresponding 2nd cell cycle fractions are 19.2%, and 20.1%, 30.6%, 25.6% and 16.0%. This indicates that the permanent treatment induces predominantly a G0/G1-arrest (inhibition of cell activation) whereas at intermediate exposure times (4 to 20 h) the PBL's escape this stringent G0/G1-block, and are arrested/slowed down in the 2nd cell cycle (inhibition of cell cycle progression).

The experimental data shown above do not formally exclude the possibility of a direct chemical reaction of CDDP and GSH in the culture medium. Therefore experiments were performed with a time-delayed addition of GSH to CDDP treated PBL's. The cell cycle distributions of the 66 h harvests applying the high-resolution BrdU/Hoechst flow cytometric analysis are shown in Figure 4. In the control culture (panel a) 38.1% of the total population remained in the G0/G1-phase, and most of the cells moved into the 2nd (G1', S', G2/M') and 3rd cell cycle (G1'', S'', G2-M''). In the CDDP treated culture (3.0 μg ml$^{-1}$) most of the cells are arrested in the G0/G1-phase, and only 14.1% moved into early S-phase (panel b). In addition the nuclear debris signals increased significantly indicating massive cell death in the CDDP treated cell culture.

**Table II** Changes of absolute cell numbers of viable and dead cells after CDDP and GSH treatment

| *GSH (mg ml$^{-1}$)* | *CDDP (μg ml$^{-1}$)* | *Cell number (× $10^5$ ml$^{-1}$)* Viable | Dead |
|---|---|---|---|
| – | – | 10.5 ± 0.7 | 0.8 ± 0.3 |
| 1.5 | – | 10.7 ± 0.5 | 0.6 ± 0.2 |
| – | 3.0 | 1.7 ± 0.2 | 1.2 ± 0.2 |
| 1.5 | 3.0 | 8.8 ± 0.3 | 0.7 ± 0.2 |

Human PBL's were inoculated at $3 \times 10^5$ cells ml$^{-1}$ and harvested 72 h after PHA activation. After trypan blue staining viable and dead cell numbers were counted in a hematocytometer (data represent mean values and SD of triplicate cultures).

**Figure 4** Cell kinetic effects of the time-delayed addition of exogenous GSH to CDDP treated human PBL's analysed by the high resolution BrdU/Hoechst-PI cell cycle technique (1st cycle: G0/G1, S, G2/M; 2nd cycle: G1', S', G2/M'; 3rd cycle: G1", S", G2/M"). Panels **a** and **b** display untreated, and CDDP treated (3.0 $\mu g\, ml^{-1}$) control cultures, respectively. Note missing cycling cells in the CDDP treated control culture in panel **b**. Panels **c**, **d**, **e**, and **f** represent cultures treated with GSH (1.5 $mg\, ml^{-1}$) immediately, 1, 4 and 20 h after CDDP toxification.

In Figure 4 panel c the BrdU/Hoechst pattern shows that the immediate addition of GSH restores the normal cell proliferative pattern. This effect is also found when GSH is given 1 and 4 h after CDDP toxification (panel d and e). If GSH is added as late as 20 h after CDDP treatment, most of the cells remain still in the 1st cycle (panel f: G0/G1, S and G2/M). However, in comparison to the CDDP treated control culture shown in panel b the PBL's have left early S-phase, and the rescue is indicated by the fact that the cells moved completely through the S- and G2/M-phase. Moreover, 2.9% of the cells have divided once, and are recognised as G1'-phase cells by the BrdU/Hoechst flow cytometric technique (panel f). The small number of 2nd cycle cells in the 20 h delay experiment is due to inhibition of PHA-induced cell activation processes by the prolonged CDDP exposure. This results in an increased lag-phase of the entry into the 1st cycle S-phase. However, higher percentages of 2nd and 3rd cell cycle cells are found at later harvest times (data not shown).

The quantitative data of the 66 h harvest are summarised in Figure 5. In comparison to the 0.3 $\mu g\, ml^{-1}$ CDDP treated culture (broken lines in panel a) at each time point the addition of GSH decreased the non-cycling G0/G1-fractions (% G0/G1 untreated control: 38.1) and S- and G2/M-populations almost to control levels. The improvement of cell proliferation due to the rescue effect increases in parallel the percentages of cells in the 2nd and 3rd cycle. The rescue pattern is also present at 1.0 $\mu g\, ml^{-1}$ CDDP (panel b). However, an incomplete rescue after a 20 h delayed addition of GSH is shown by the slight increased S-phase population (open bar, S-phase, panel b), which is paralleled by a smaller increase/rescue of the 2nd/3rd cell cycle population. At 3.0 $\mu g\, ml^{-1}$ CDDP the PBL's are completely arrested in G0/G1- and early S-phase (panel c, broken lines). The rescue effect is similar whether GSH is added immediately, 1 or 4 h after CDDP toxification. Although the percentage of the G0/G1-population after a 20 h treatment decreased closely to control levels, the S- and G2/M-phase fractions are still increased due to prolonged lag-phase duration.

*GSH/GSSG levels in medium*

In order to evaluate possible alterations of concentrations of GSH in culture medium, HPLC analysis was applied for

**Figure 5** Rescue effect of CDDP treated human PBL's after delayed addition of GSH. Cell harvest was 66 h after PHA stimulation. The data are derived from Figure 4. GSH (1.5 $mg\, ml^{-1}$) was added at culture setup, 1, 4 and 20 h after CDDP toxification. The broken lines display CDDP controls of PBL's without GSH treatment. The data represent the percentages of the G0/G1, S and G2/M phase cells in the 1st cycle, and the sum of the 2nd and 3rd cycle compartments. Panels **a**, **b** and **c**: 0.3, 1.0 and 3.0 $\mu g\, ml^{-1}$ CDDP.

quantification of reduced and oxydised glutathione. GSH was dissolved at different concentrations in PBS adjusted to pH 7.2, and HPLC analysis was 1 h later. The retention time for oxydised GSSG and reduced GSH was 8.3 and 4.7 min, respectively. The GSH-peaks of the HPLC curves were quantitated by integration of the peak area, and runs were performed at 0.015, 0.15 and 1.5 mg $ml^{-1}$ GSH. The data revealed that 87.3, 71.4 and 60.5% of the initial concentrations indicated above were present as GSH in PBS. Although analysis was not performed in medium supplemented with FCS, this approximation indicates the effective GSH concentrations in GSH supplemented RPMI-medium, and show slightly lower values due to GSH/GSSG transition.

### Discussion

Glutathione is an important multifunctional biomolecule which is of extensive interest in cancer chemotherapy (Arrick & Nathan, 1984; Mitchell *et al.*, 1989; Mistry & Harrap, 1991). In animal models and clinical studies the administration of GSH decreased nephrotoxicity and myelotoxicity (increased WBC) induced by CDDP treatment (Zunino *et al.*, 1983; Zunino *et al.*, 1989; Oriana *et al.*, 1987; Di Re *et al.*, 1990). We investigated whether CDDP treated human PBL's can be protected by extracellular applied GSH in order to explain partially the increased WBC observed in clinical studies (Oriana *et al.*, 1987; Di Re *et al.*, 1990). Most of the CDDP related cell kinetic studies performed used permanent cell lines as *in vitro* model systems (Salles *et al.*, 1983; Kanno *et al.*, 1985; Sorenson & Eastman, 1988; Fujikane *et al.*, 1989). However, for the investigation of severe cellular side effects induced by clastogens *in vivo* (Canetta *et al.*, 1985; Mangioni *et al.*, 1989), normal diploid cells are the more adequate cell systems.

For detailed analysis we applied a novel flow cytometric cell kinetic analysis (BrdU/Hoechst-PI technique) that is characterised by its vastly improved information about cell proliferation of heterogenous cell systems (Kubbies *et al.*, 1987; Kubbies *et al.*, 1989) and its much lower statistical variation in repeat experiments (Rabinovitch, 1983). We show that multiple cell proliferative disturbances are induced in PBL's by CDDP. Cell activation (G0/G1) as well as cell cycle progression (S and G2/M) are inhibited in PBL's after PHA-activation. At lower CDDP concentrations the cell cycle progression inhibition in S- and G2/M is more prominent, whereas at higher doses the cell activation process and early S-phase cell cycle progression are affected. The doses of CDDP used in our experiments correspond to the range of concentrations found in the plasma of CDDP treated animals (Pfeifle *et al.*, 1985; Goel *et al.*, 1989; Sasaki *et al.*, 1989). Therefore similar cell kinetic disturbances might be anticipated in *in vivo* activated peripheral blood cells.

Treatment of cells exposed to CDDP with exogenous reduced GSH abolishes cell proliferative disturbances: (a) the activation process is no longer severely affected as indicated by normal lag-phase durations and lower percentages of non-activated G0/G1 cells, and (b) the S- and G2/M-phase arrest and/or slow down disappeared as shown by normal 1st, 2nd and 3rd cell cycle distributions (Figure 2, Table I). A complete rescue is observed only at the highest GSH concentration of 1.5 mg $ml^{-1}$ (equivalent to 4.9 mM) which is non-toxic to human PBL's (see also Table II for absolute cell numbers). This is a 500-fold excess of extracellular GSH in comparison to the highest dose of CDDP. However, this value is comparable to intracellular GSH concentrations of 3 to 5 mM in human leukocytes (Kosover, 1978). In addition the effective exogenous GSH concentrations applied to whole cells in our studies is in accordance with the GSH concentration necessary to stop conversion of short-lived CDDP-DNA-monoaduct to bifunctional adducts on isolated DNA (Eastman, 1987). These more long-lived GSH/CDDP/DNA-monoadducts might be inactivated by DNA-repair mechanisms. Therefore it is conceivable that the complete GSH rescue as late as 4 h after CDDP treatment might be explained by molecular reaction of GSH with such short-lived CDDP/DNA-monoadducts.

The time-delayed addition of GSH (1–20 h) to CDDP treated cells should exclude the formal possibility of a significant direct interaction between both agents in the culture medium. As shown in Figures 4 and 5, a 1 to 4 h delay of the addition of GSH shows a complete rescue effect. Although a 20 h delay is still effective less PBL's are found in the 2nd and 3rd cell cycle. This is simply due to the increased lag-phase duration of PHA activated PBL's induced by a 20 h CDDP treatment (delay of cell activation), and due to the washing procedure which removes the cell cycle progression factor IL-2 secreted from T-cells. It has been shown previously that (a) maximum saturation of CDDP-DNA adducts is in the range of 4 to 8 h (Eastman, 1987; Roberts & Friedlos, 1987), and (b) a 1 to 4 h delay of the administration of cysteamine and diethyldithiocarbamate *in vitro* still protects cells from CDDP toxicity (Bodenner *et al.*, 1986; Shrieve & Harris, 1982). Our experimental results are in accordance with these data, and demonstrate that the CDDP/GSH rescue effect obviously represents a real biological phenomenon.

Preliminary experiments for intracellular quantitation of GSH using the flow cytometric monochlorobimane technique indicate that CDDP treatment of PHA-stimulated PBL's with extracellular GSH restores normal intracellular GSH and protein thiol content (data not shown). Previously it has been demonstrated that permanent human lymphoid cell lines are not permeable to extracellular GSH (Wellner *et al.*, 1984). Our own experimental evidence applying BSO in PBL's as an inhibitor of intracellular GSH synthesis also gave no evidence for the transport of extracellular GSH into the cells. Recent experiments suggest that glutathione ester as cell permeant molecules might decrease lethal cis-platinum toxicity in mice. However the effective dose was only 2- to 5-fold lower in comparison to GSH (Anderson *et al.*, 1990). However, GSH ester is taken up by all cells, and it remains to be shown whether there is selective chemoprotection or cytotoxicity on tumours or tumour cells in comparison to normal diploid cells or tissue *in vivo* (Zunino *et al.*, 1983; Zunino *et al.*, 1989; Oriana *et al.*, 1987; Di Re *et al.*, 1990).

At the present we can only suggest that increased extracellular GSH levels might promote intracellular GSH-synthesis for example via increased, normally GSH-synthesis rate-limiting cyteine uptake, although at the present the intracellular effects remain obscure at the molecular level. Flow cytometric studies of intracellular GSH/thiol content in single cells applying other SH-biomolecules and inhibitors of GSH synthesis should give further insights into cellular and molecular pathways of the rescue from CDDP toxicity.

We thank Dr H. Prinz for GSH/GSSG quantification by HPLC analysis, K. Bauer for technical support.

### References

ANDERSON, M.E., NAGANUMA, A. & MEISTER, A. (1990). Protection against cis-platin toxicity by administration of glutathione ester. *FASEB J.*, **4**, 3251.

ARRICK, B.A. & NATHAN, C.F. (1984). Glutathione metabolism as a determinant of therapeutic efficacy: a review. *Cancer Res.*, **44**, 4224.

BODENNER, D.L., DEDON, P.C., KENG, P.C., KATZ, J.C. & BORCH, R.F. (1986). Selective protection against cis-diamminedichloroplatinum (II)-induced toxicity in kidney, gut, and bone marrow by diethyldithiocarbamate. *Cancer Res.*, **46**, 2751.

BORCH, R.F., KATZ, J.C., LIEDER, P.H. & PLEASANTS, M.E. (1980). Effect of diethyldithiocarbamate rescue on tumor response to cis-platinum in a rat model. *Proc. Natl Acad. Sci.*, **77**, 5441.

CANETTA, R., ROSENCWEIG, M. & CARTER, S.K. (1985). Carboplatin: the clinical spectrum to date. *Cancer Treat. Rev.*, **12**, 125.

COMBRIER, E., METEZEAU, P., RONOT, X., GACHELIN, H. & ADOLPHE, M. (1989). Flow cytometric assessment of cell viability: a multifaceted analysis. *Cytotechnology*, **2**, 27.

DI RE, F., BOEHM, S., ORIANA, S., SPATTIE, G.B. & ZUNINO, F. (1990). Efficacy and safety of high-dose cis-platin and cyclophosphamide with glutathione protection in the treatment of bulky advanced epithelial ovarian cancer. *Cancer Chemother. Pharmacol.*, **25**, 355.

EASTMAN, A. (1987). Cross-linking of glutathione to DNA by cancer chemotherapeutic platinum coordination complexes. *Chem. Biol. Interations*, **61**, 241.

FILIPSKI, J., KOHN, K.W., PRATHER, R. & BONNER, W. (1979). Thiourea reverses cross-linked and restores biological activity in DNA treated with dichlorodiamminoplatinum. *Science*, **204**, 181.

FUJIKANE, T., SHIMIZU, T., TSUJI, T., ISHADA, S. & OHSAKI, Y. & ONODERA, S. (1989). Flow cytometric analysis of the kinetic effects of cis-platin on lung cancer cells. *Cytometry*, **10**, 788.

GANDARA, D.R., PEREZ, E.A., WIEBE, V. & DE GREGORIO, M.W. (1991). Cis-platin chemoprotection and rescue: pharmacologic modulation of toxicity. *Sem. Oncol.*, **18**, 49.

GOEL, R., CLEARY, S.M., HORTON, C. & 4 others (1989). Effect of sodium thiosulfate on the pharmacokinetics and toxicity of cisplatin. *J. Natl Cancer Inst.*, **81**, 1552.

GRINGERI, A.G., KENG, P.C. & BORCH, R.F. (1988). Diethyldithiocarbamate inhibition of murine bone marrow toxicity caused by cis-diamminedichloroplatinum(II) or diammine(1,1-cyclobutandicarboxylato)platinum(II). *Cancer Res.*, **48**, 5708.

KANNO, S., HYODO, M., SUZUKI, K. & OHKIDO, M. (1985). Effect of DNA-damaging agents on DNA replication and cell cycle progression of cultured mouse mammary carcinoma cells. *Jpn. J. Cancer Res.*, **76**, 289.

KOSOVER, N.S. (1978). The glutathione status of cells. *Int. Rev. Cytol.*, **54**, 109.

KUBBIES, M., SCHINDLER, D., HOEHN, H., FRIEDL, R. & RABINOVITCH, P.S. (1987). BrdU/Hoechst cell cycle analysis applied to Fanconi anemia and inhibitory agents. In *Clinical Cytometry and Histometry*, Burger, G., Ploem, J.S. & Goerttler, K. (eds), p. 243, Academic Press, Inc.: London.

KUBBIES, M., HOEHN, H., SCHINDLER, D., CHEN, Y.C. & RABINOVITCH, P.S. (1989). Cell cycle analysis via BrdU-Hoechst flow cytometry: principles and applications. In *Flow Cytometry: Advanced Research and Clinical Applications*, Vol. II, Yen, A., (ed.), p. 5, CRS-Press: Boca Raton, FL.

KUBBIES, M., FRIEDL, R., KOEHLER, J., RABINOVITCH, P.S. & HOEHN, H. (1990*a*). Improvement of human lymphocyte proliferation and alteration of Il-2 secretion kinetics by alpha-thioglycerol. *Lymphokine Res.*, **9**, 95.

KUBBIES, M. (1990*b*). Flow cytometric recognition of clastogen induced chromatin damage in G0/G1 lymphocytes by non-stoichiometric Hoechst fluorochrome binding. *Cytometry*, **11**, 386.

LAZO, J.S. & BAHNSON, R.R. (1989). Pharmacological modulators of DNA-interactive antitumor drugs. *Trends Pharm. Sci.*, **10**, 369.

LIPPARD, S.J. (1982). New chemistry of an old molecule: cis[Pt-$(NH_3)_2Cl_2$]. *Science*, **218**, 1075.

MANGIONI, C., BOLIS, G., PECORELLI, S. & 10 others (1989). Randomized trial in advanced ovarian cancer comparing cisplatin and carboplatin. *J. Natl Cancer Inst.*, **81**, 1464.

MARKMAN, M., CLEARY, S., PFEIFLE, C.E. & HOWELL, S. (1985). High-dose intracavitary cisplatin with intravenous thiosulfate. *Cancer*, **56**, 2364.

MEISTER, A. & ANDERSON, M.E. (1983). Glutathione. *Ann. Rev. Biochem.*, **52**, 711.

MISTRY, P. & HARRAP, K.R. (1991). Historical aspects of glutathione and cancer chemotherapy. *Pharmac. Ther.*, **49**, 125.

MITCHELL, J.B., COOK, J., DE GRAFF, W., GLATSTEIN, E. & RUSSO, A. (1989). Keynote address: glutathione modulation in cancer treatment: will it work. *Int. J. Radiat. Oncol. Biol. Phys.*, **16**, 1289.

ORIANA, S., BOEHM, S., SPATTIE, G.B., ZUNINO, F. & DI RE, F. (1987). A preliminary clinical experience with reduced glutathione as protector against cisplatin-toxicity. *Tumori*, **73**, 337.

PFEIFLE, C.E., HOWELL, S.B., FELTHOUSE, R.D. & 4 others (1985). High-dose cisplatin with sodium thiosulfate protection. *J. Clin. Oncol.*, **3**, 237.

RABINOVITCH, P.S. (1983). Regulation of human fibroblast growth rate by both non-cycling cell fraction and transition probability is shown by growth in 5-BrdU followed by Hoechst 33258 flow cytometry. *Proc. Natl Acad. Sci.*, **80**, 2951.

ROBERTS, J.J. & FRIEDLOS, F. (1987). Quantitative estimation of cis-platin induced DNA interstrand cross-links and their repair in mammalian cells: relationship to toxicity. *Pharmacol. Ther.*, **34**, 215.

SALLES, B., BUTOUR, J.L., LESCA, C. & MACQUET, J.P. (1983). Cis-$Pt(NH_3)_2Cl_2$ and trans-$Pt(NH_3)_2Cl_2$ inhibit DNA synthesis in cultured L1210 leukemia cells. *Biochem. Biophys. Res. Comm.*, **112**, 555.

SASAKI, Y., FUKUDA, M., FUJIWARA, Y., TAMURA, T., EGUCHI, K. & SHINKAI, T. (1989). Pharmacokinetic study of carboplatin in comparison with cis-platin. *Chemotherapy*, **37**, 280.

SHRIEVE, D.C. & HARRIS, J.W. (1982). Protection against cis-dichloro-diammine Pt(II) cytotoxicity *in vitro* by cysteamine. *Int. J. Radiat. Oncol. Biol. Res.*, **8**, 585.

SORENSON, C.M. & EASTMAN, A. (1988). Mechanism of cis-diamminedichloroplatinum(II)-induced cytotoxicity: role of G2 arrest and DNA double strand breaks. *Cancer Res.*, **48**, 4484.

WELLNER, V.P., ANDERSON, M.E., RAJINDER, N.P., JENSEN, G.L. & MEISTER, A. (1984). Radioprotection by glutathione ester: transport of glutathione ester into human lymphoid cells and fibroblasts. *Proc. Natl Acad. Sci.*, **81**, 4732.

ZUNINO, F., TOFANETTI, O., CAVALLETTI, E. & SAVI, G. (1983). Protection effect of reduced glutathione against cis-dichloro-diammine-platinum(II)-induced nephrotoxicity and lethal toxicity. *Tumori*, **69**, 105.

ZUNINO, F., PRATESI, G., MICHELONI, A, CAVALLETTI, E., SALA, F. & TOFANETTI, O. (1989). Protective effect of reduced glutathione against cisplatin-induced renal and systemic toxicity and its influence on the therapeutic activity of the antitumor drug. *Chem. Biol. Interations*, **70**, 89.

ZWELLING, L.A., FILIPSKI, J. & KOHN, K.W. (1979). Effect of thiourea on survival and DNA-cross-link formation in cells treated with platinum (II) complexes, L-phenylalanine mustard, and bis-(2-chloroethyl)methylamine. *Cancer Res.*, **39**, 4989.